# Supplementary material for: Seminoma and Embryonal Carcinoma Footprints Identified by Analysis of Integrated Genome-Wide Epigenetic and Expression Profiles of Germ Cell Cancer Cell Lines
Source: PLoS One. 2014 Jun 2;9(6):e98330. doi: 10.1371/journal.pone.0098330 (PMC4041891; doi:10.1371/journal.pone.0098330)
Supplement: File S1 — ZIP file containing DMRforPairs output for significant regions. Please start from the html files. (ZIP) [file pone.0098330.s008.zip › figures/26504.pdf]

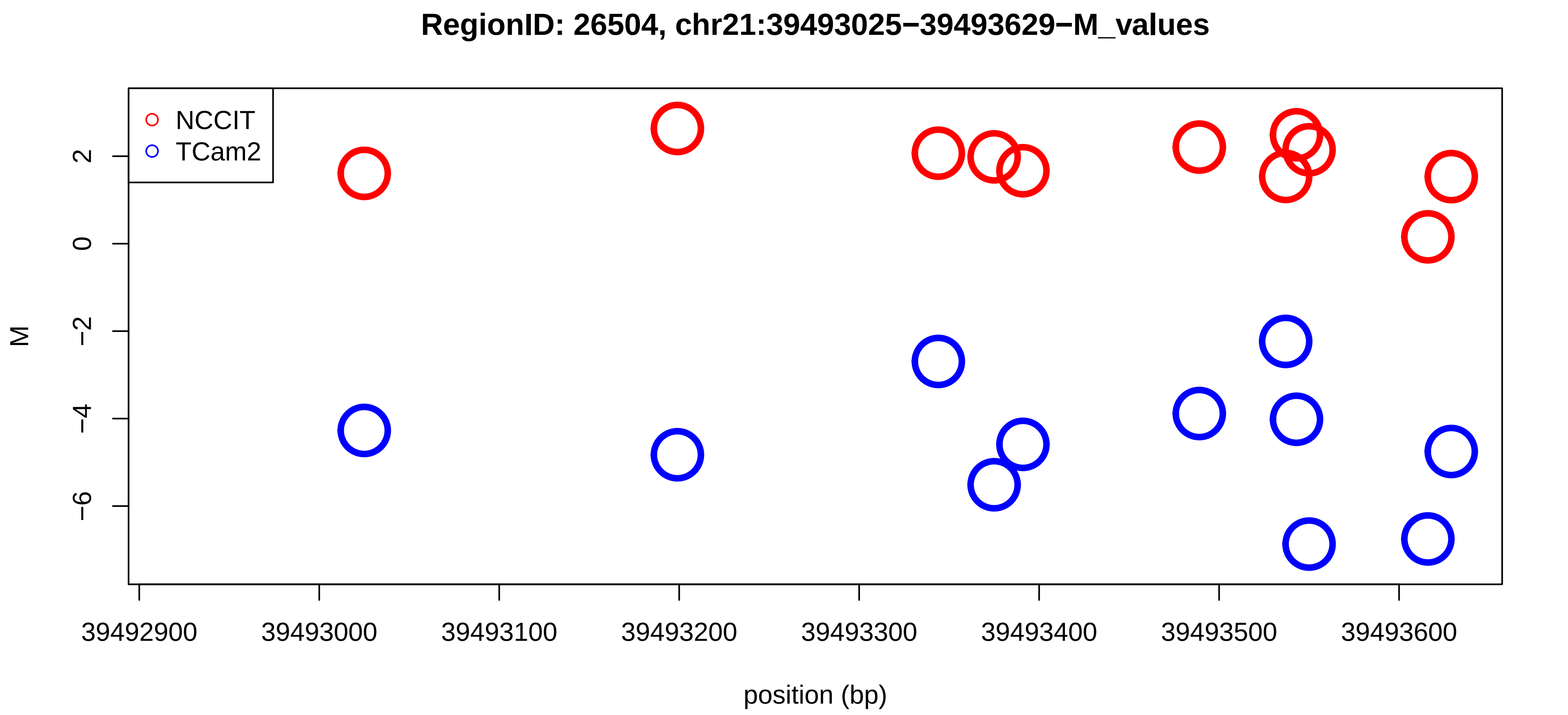
RegionID: 26504, chr21:39493025−39493629−M_values
NCCIT
TCam2
M
2
0
−2
−4
−6
39492900
39493000
39493100
39493200
39493300
39493400
39493500
39493600
position (bp)

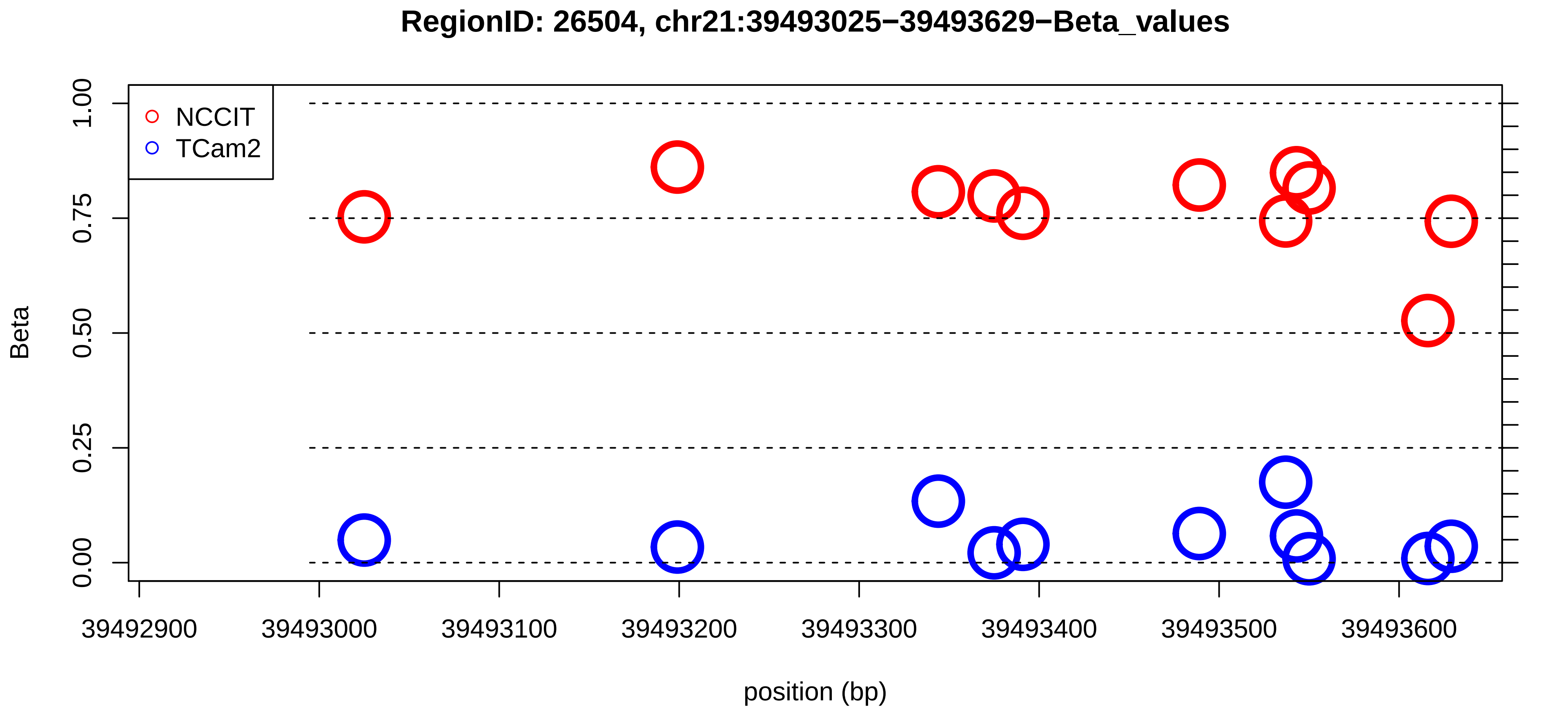
RegionID: 26504, chr21:39493025−39493629−Beta_values
NCCIT
TCam2
Beta
1.00
0.75
0.50
0.25
0.00
39492900
39493000
39493100
39493200
39493300
39493400
39493500
39493600
position (bp)

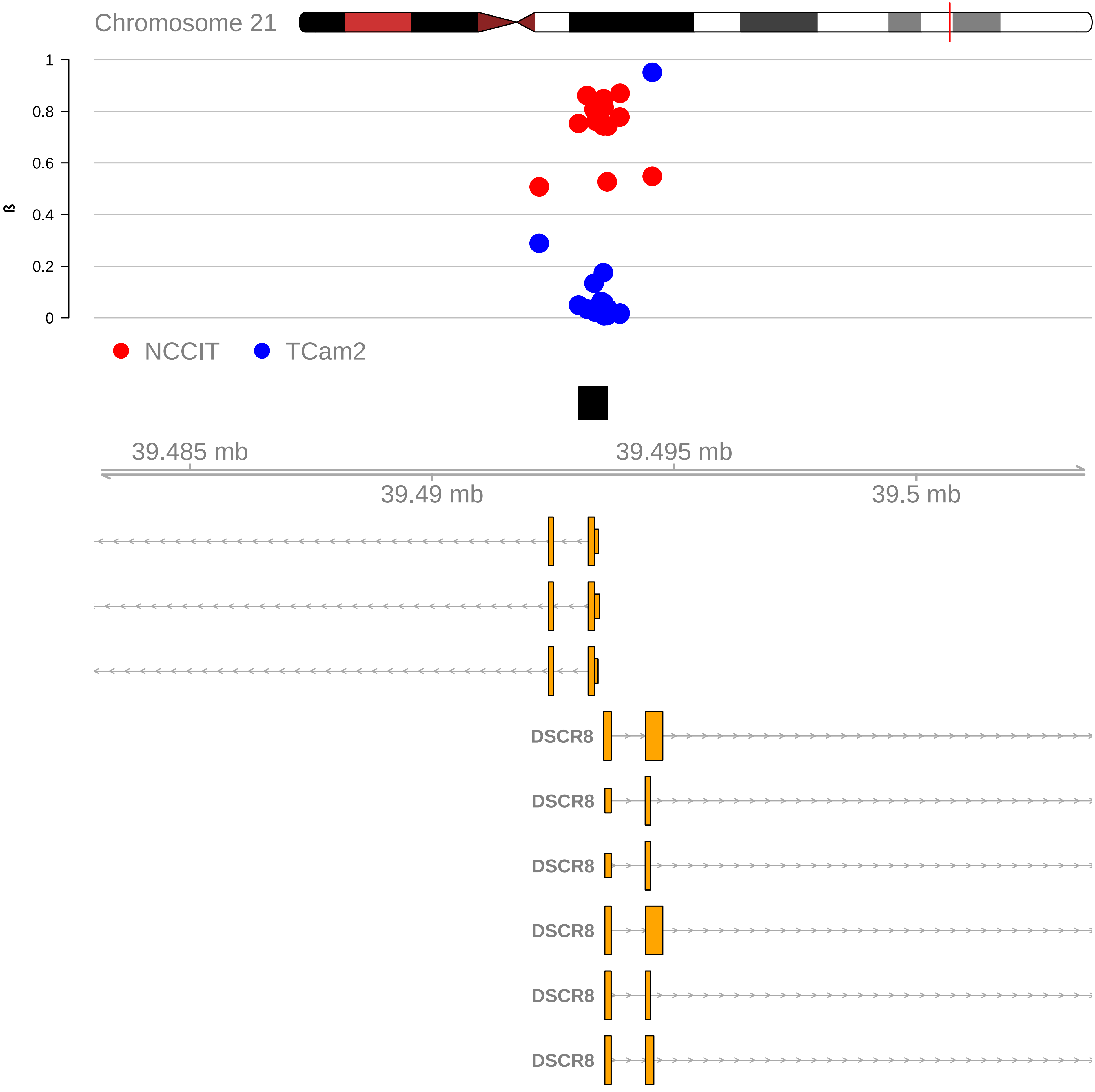

Chromosome 21
β
1
0.8
0.6
0.4
0.2
0
NCCIT
TCam2
39.485 mb
39.495 mb
39.49 mb
39.5 mb
DSCR8
DSCR8
DSCR8
DSCR8
DSCR8
DSCR8
